# First clinical experience with the Kora pacemaker system in congenital complete heart block in newborn infants

**DOI:** 10.1186/s12887-019-1494-7

**Published:** 2019-04-24

**Authors:** Stefan Kurath-Koller, Sabrina Schweintzger, Gernot Grangl, Ante Burmas, Andreas Gamillscheg, Martin Koestenberger

**Affiliations:** Division of Pediatric Cardiology, Department of Pediatrics, Medical University Graz, Auenbruggerplatz 34/2, A-8036 Graz, Austria

**Keywords:** Pacemaker, Children, Congenital heart block, Cardiac output

## Abstract

**Background:**

To report first clinical experience on three cases of congenital complete heart block and the use of a pacemaker system with a maximum lower rate interval of 95 beats per minute.

**Methods:**

We retrospectively analyzed three patients treated with a pacemaker system with a maximum lower rate interval of 95 beats per minute suffering from congenital complete heart block. We report a follow up period of 2.9 years, focusing on the patients’ growth, development, and adverse events, as well as pacemaker function.

**Results:**

In all three patients pacemaker function was impeccable, including minute ventilation sensor rate adaption. All patients showed limited growths as expected, adequate development, good feeding tolerability and circadiane heart rate adaption. One patient experienced skin traction and revision. All patients showed high aortic velocity time integral values after birth.

**Conclusion:**

The use of a pacemaker system with a maximum lower rate interval of 95 beats per minute in infants suffering from congenital complete heart block and showing high aortic VTI values seems to be feasible and to result in limited growths but adequate development.

**Electronic supplementary material:**

The online version of this article (10.1186/s12887-019-1494-7) contains supplementary material, which is available to authorized users.

## Background

Indications for pacemaker therapy in the neonatal population are rare and include e.g. congenital complete heart block (CCHB) [1]. In the neonatal population, the size of the pacemaker system is of interest, because space for implantation is scarce due to physiological conditions. The pacemaker system we used (Kora pacemaker system, MicroPort, formerly LivaNova PLC, London, UK) currently represents one of the smallest pacemaker systems available comprising 8 cc. This pacemaker has been demonstrated to be safe and effective in adults [2]. Its’ longevity and size place it at special interest in patients below 5 kg of body weight. However, it has been questioned whether this pacemaker system is able to cover for neonatal use due to the fact that the lower rate interval (LRI) in this single chamber system is limited to 630 ms (95 beats per minute (bpm)). Given normal heart rates of neonates of approximately 140 bpm, this might be too low for an acceptable level of life quality and growing capacity. The heart minute volume adaption of infants is mainly controlled by frequency and only marginal by stroke volume due to physiological conditions [3]. So far no clinical data on its use in neonates with CCHB are available.

## Hypothesis

We hypothesize that an LRI of 630 ms allows for adequate growths and development in newborns and infants suffering from CCHB, as cardiac output adaption took place prenatally.

## Methods

We performed a systematic literature research on the use of the Kora pacemaker system in neonates using PUBMED, and MEDLINE databases. To the best of our knowledge, we are the first to report on 3 infants in whom a single chamber pacemaker system with an LRI of 630 ms was implanted. We report a follow up period of 2.9 years, focusing on the patients’ growth, feeding behavior and development, as well as pacemaker function, parameters, heart rate modulation and function of minute ventilation sensors. Furthermore adverse events and tolerability of the pacemaker system were reviewed. The following conditions were considered adverse events: infection of the implantation site, skin traction, suture dehiscence, lead breakage/dislocation, development of scar tissue resulting in elevation of threshold levels. For assessment of growth World Health Organization growth charts were used. Pacemaker parameters and minute ventilation sensor function and activity were analyzed using follow up protocols saved in our database. Protocols were assessed regarding programmed mode, upper rate interval, intrinsic rhythm, impedance, threshold, amplitude, impulse duration, circadian heart rate distribution and rate adaption during crying and feeding. Furthermore we assessed echocardiography records regarding aortic velocity time integral as an estimator on cardiac output.

## Results

Demographics on included patients are given in Table 1. We report on a follow up period of 2.3 to 2.9 years. One male and two female newborns had prenatal heart block and prenatal heart rates of about 57 bpm. The pacemakers were implanted at 2, 14 and 142 days, respectively, of age with an average weight of 3.1 kg at time of implantation. Lead positioning was epicardial and the device was placed abdominally in all three patients. Postnatal echocardiographic findings showed that cardiac output was adjusted to the circumstances with aortic velocity time integral (VTI) levels of about 24 cm, resembling values usually found in 7 years old children [4] (Table 1a).

**Table 1:** Demographic data on infants with Kora pacemaker system

	Patient 1	Patient 2	Patient 3
a) Demographic Data			
Sex	F	F	M
GA (wks + d)	37 + 0	36 + 4	38 + 0
BW (kg)	2,8	3	2,9
APGAR	7/8/9	8/8/8	7/8/9
Fetal HR (bpm)	50	64	54
pp HR (bpm)	55	60	52
pp AoVTI (cm)	26,1	21,8	23,9
Age at Implant (d)	2	142	14
Weight at Implant (kg)	2,8	5,1	3,1
Follow Up (a)	2,9	2,2	2,3
Echocardiography	unremarkable	unremarkable	unremarkable
b.) Initial programming			
Model	Kora 100 SR	Kora 100 SR	Kora 250 SR
Mode	VVIR	VVIR	VVIR
Lower rate interval	90	95	90
Intrinsic rhythm (bpm)	50	69	58
Polarity	unipolar	unipolar	bipolar
Impedance (Ω)	341	510	900
R-wave (mV)	7,9	7,75	15
Threshold (V/ms)	1,5/0,5	1,75/0,85	0,75/0,35
Impulse (ms)	0,5	0,85	0,35
Amplitude (V)	4	3,5	3

Leg.: *F* female, *M* male, *GA* gestational age, *a* years, *wks* weeks, *d* days, *kg* kilogram, *bpm* beats per minute, *Ω* Ohm, *mV* millivolt, *ms* milliseconds, *V* Volt, *pp* post partum, *AoVTI* aortic velocity time integral, *HR* heart rate, *Implant* Implantation

**Fig. 1:**
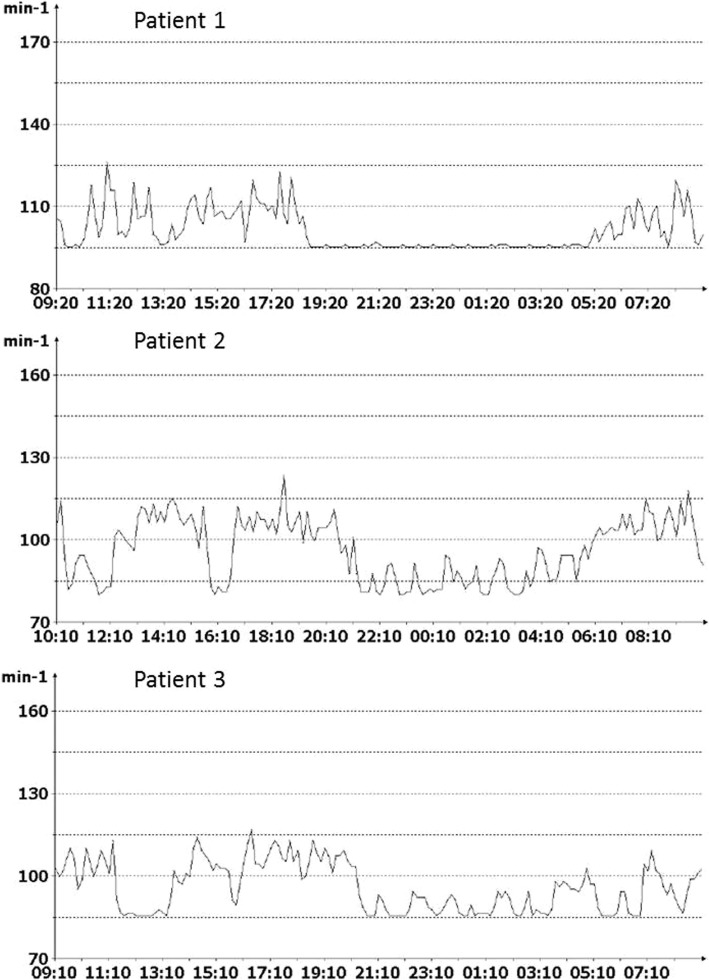
Circadian heart rate diagrams of our patients. Legend: x-axis shows time of day as hh:mm; y-axis shows heart rate as beats per minute. Diagrams were recorded at 2.5, 2 and 1.9 years respectively

Except for skin traction, which resulted in suture dehiscence and pacemaker revision in one newborn, no adverse events occurred. All devices were programmed in VVIR mode at the lower rate interval of 630 ms. Initial programming specifics are given in Table 1b. Pacemaker follow up were performed at short term interval throughout hospital stay following implantation and was loosened gradually to a now 6-month interval. Throughout the follow up period threshold levels, amplitude, impedance and minute ventilation sensor function were unremarkable. Minute ventilation sensor function resulted in circadian heart rate adaption and adequate rate response during feeding or crying (Fig. 1). Accelerometer sensors were not used. Clinically, all neonates improved remarkably throughout follow up and grew along their centile curve (Additional file 1: Figure S1, Additional file 2: Figure S2, Additional file 3: Figure S3). All three infants were at the 10th percentile at birth and continued to grow along the 10th percentile curve with low dropping along time. Feeding tolerability of age related adequate volumes of milk and later on solid foods was excellent in all patients. Motor and cognitive development was unimpaired in all patients as assessed using Denver Developmental Screening Test (Additional file 4: Figure S4, Additional file 5: Figure S5, Additional file 6: Figure S6).

## Discussion

We report on the first clinical experience with a single chamber pacemaker system limited to an LRI of 630 ms in neonates. Due to the limited LRI in this single chamber system, concern has been raised whether it would suffice for neonates to contain adequate cardiac minute volume and growth. As we detected high values of aortic VTI (cardiac output marker) in immediate postnatal echocardiography studies, we assumed that cardiac output compensation was sufficient to use a maximum LRI of 95 bpm. Cardiac output compensation seems to have evolved prenatally due to low fetal heart rates. Along follow up VTI values remained constant. We hoped that the smallness of the device would aid in regard of a better implantation outcome and tolerability concerning skin traction and suture dehiscence. Furthermore we figured an MV sensor for rate response being a good choice in infants. Despite using such a small system suture dehiscence accounted for a complication rate of 33%. The function of MV sensors in children with pacemakers has been shown by Cabrera et al. [5]. Neonates and young infants are at need of high heart rates to meet cardio-circulatory demands when crying or drinking. None of these events would trigger rate adaption via accelerometer sensors since the patients do not move or accelerate. However, most infants may be rocked to calm or sleep. Using accelerometer sensors, this might trigger an inappropriate rise in heart rate. The MV sensor may aid pacemaker function in neonates in terms of better rate adaption compared to accelerometer sensors. However, it seems important that there is no evidence of MV sensor function with epicardial leads. In general, infants’ cardiac minute volume is mainly heart rate dependent since inotropic capacity is impaired throughout this period of life. Never the less, our infants showed VTI values usually found in 7 years old children. We hypothesize that this prenatal adaption in cardiac output due to low prenatal heart rates, enables 95 bpm to be sufficient to meet cardio-circulatory requirements in neonates.

## Conclusion

A limited lower rate interval of 630 ms seems to suffice adequate development along with limited growth in infants suffering from CCHB, given a sufficient adaption in neonatal stoke volume. Size of the pacemaker system, however, seems crucial in regard of complications like skin-traction and surgical revision. Never the less, children remain poor cousins in cardiac device therapy. Small sized pacemaker systems meeting pediatric needs in terms of rate limitation and implemented sensors are warranted, especially for the neonatal population. However, such devices are still lacking.

## Additional files

Additional file 1: **Figure S1.** Growth centile curves of patient 1. Legend: x-axis shows age in months, y-axis shows body weight in kilogram on the right lower side and body lengths in cm on the left and right upper side. (JPG 930 kb)

Additional file 2: **Figure S2.** Growth centile curves of patient 2. Legend: x-axis shows age in months, y-axis shows body weight in kilogram on the right lower side and body lengths in cm on the left and right upper side. (JPG 931 kb)

Additional file 3: **Figure S3.** Growth centile curves of patient 3. Legend: x-axis shows age in months, y-axis shows body weight in kilogram on the right lower side and body lengths in cm on the left and right upper side. (JPG 935 kb)

Additional file 4: **Figure S4.** Denver Developmental Screening Test results of patient 1. (PDF 198 kb)

Additional file 5: **Figure S5.** Denver Developmental Screening Test results of patient 2. (PDF 202 kb)

Additional file 6: **Figure S6.** Denver Developmental Screening Test results of patient 3. (PDF 198 kb)

## Abbreviations

bpm: Beats per minute
CCHB: Congenital complete heart block
LRI: Lower rate interval
MV: Minute ventilation
VTI: Velocity time integral
