# Supplementary figures and images for: First clinical experience with the Kora pacemaker system in congenital complete heart block in newborn infants
Source: BMC Pediatr. 2019 Apr 24;19:124. doi: 10.1186/s12887-019-1494-7 (PMC6480703; doi:10.1186/s12887-019-1494-7)

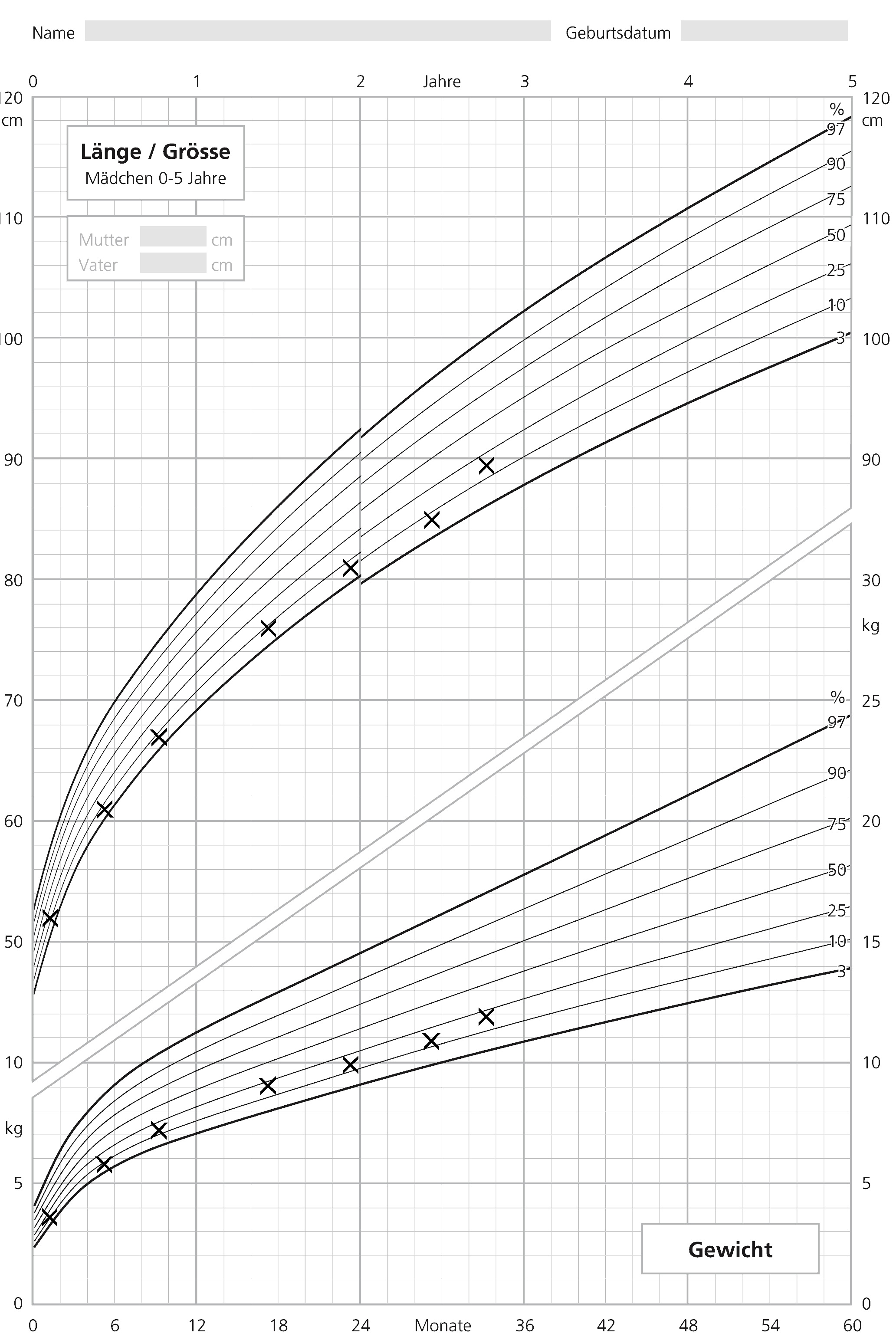

Supplement: Supplementary file 1 — Figure S1. Growth centile curves of patient 1. Legend: x-axis shows age in months, y-axis shows body weight in kilogram on the right lower side and body lengths in cm on the left and right upper side. (JPG 930 kb) [file 12887_2019_1494_MOESM1_ESM.jpg]

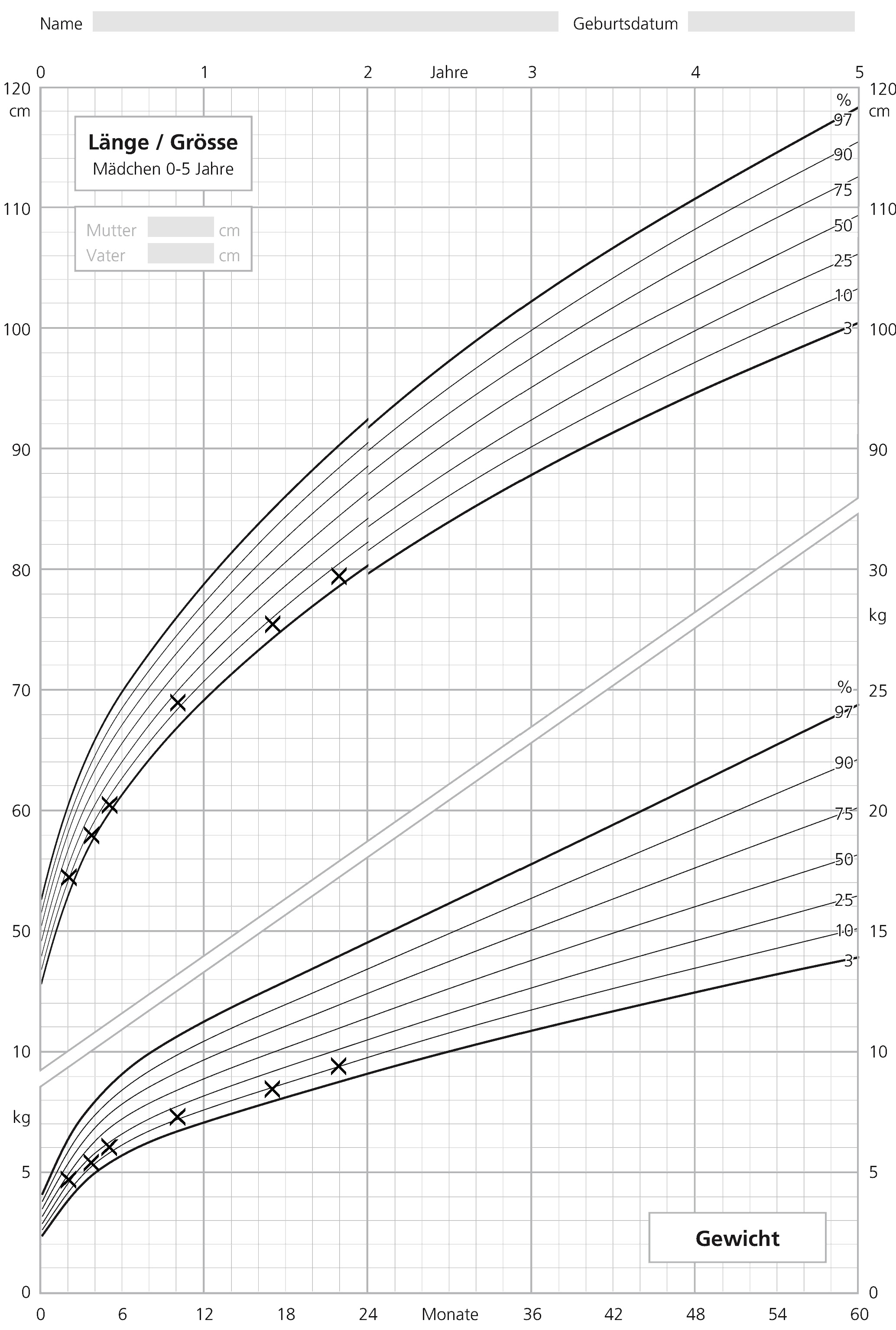

Supplement: Supplementary file 2 — Figure S2. Growth centile curves of patient 2. Legend: x-axis shows age in months, y-axis shows body weight in kilogram on the right lower side and body lengths in cm on the left and right upper side. (JPG 931 kb) [file 12887_2019_1494_MOESM2_ESM.jpg]

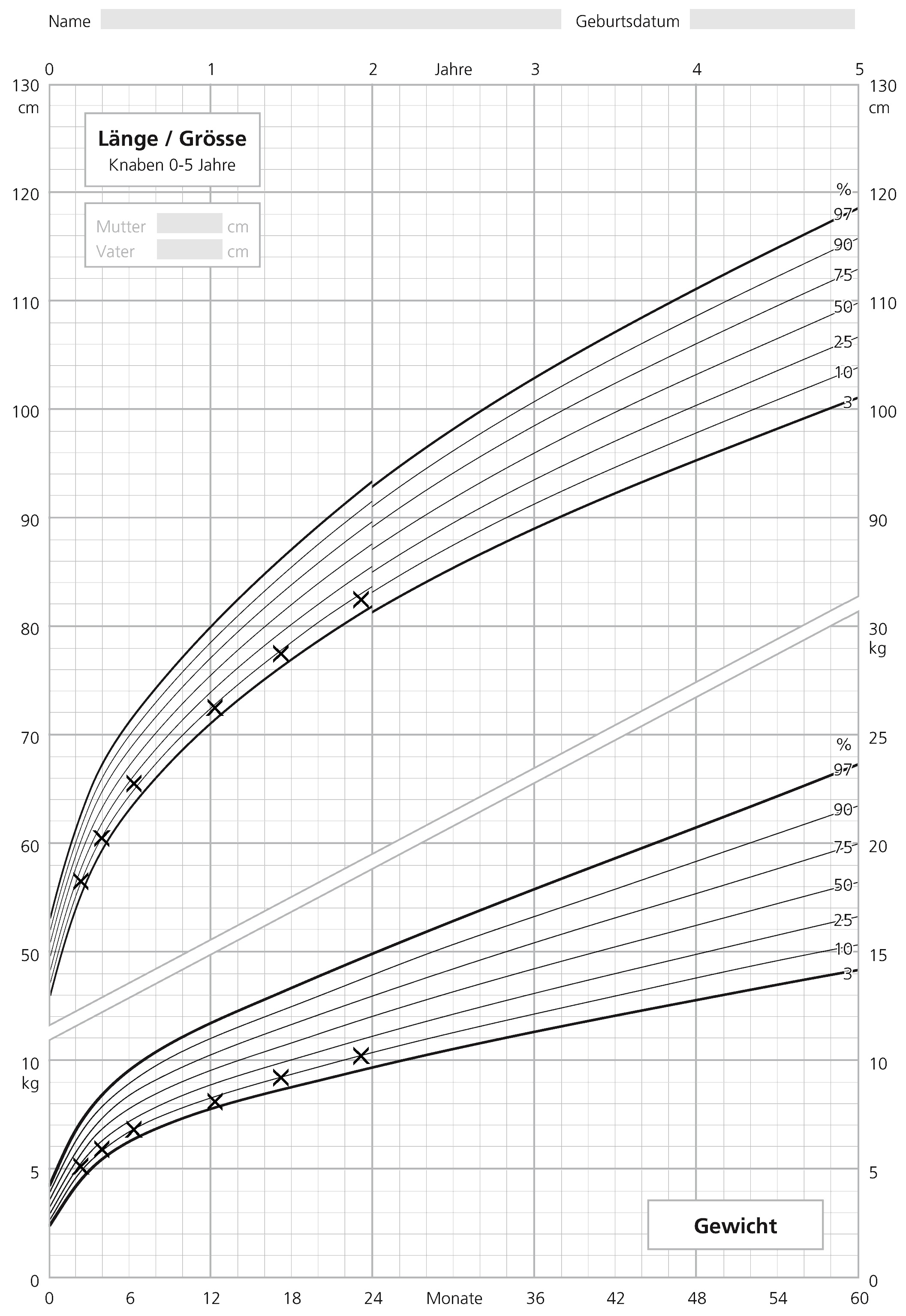

Supplement: Supplementary file 3 — Figure S3. Growth centile curves of patient 3. Legend: x-axis shows age in months, y-axis shows body weight in kilogram on the right lower side and body lengths in cm on the left and right upper side. (JPG 935 kb) [file 12887_2019_1494_MOESM3_ESM.jpg]

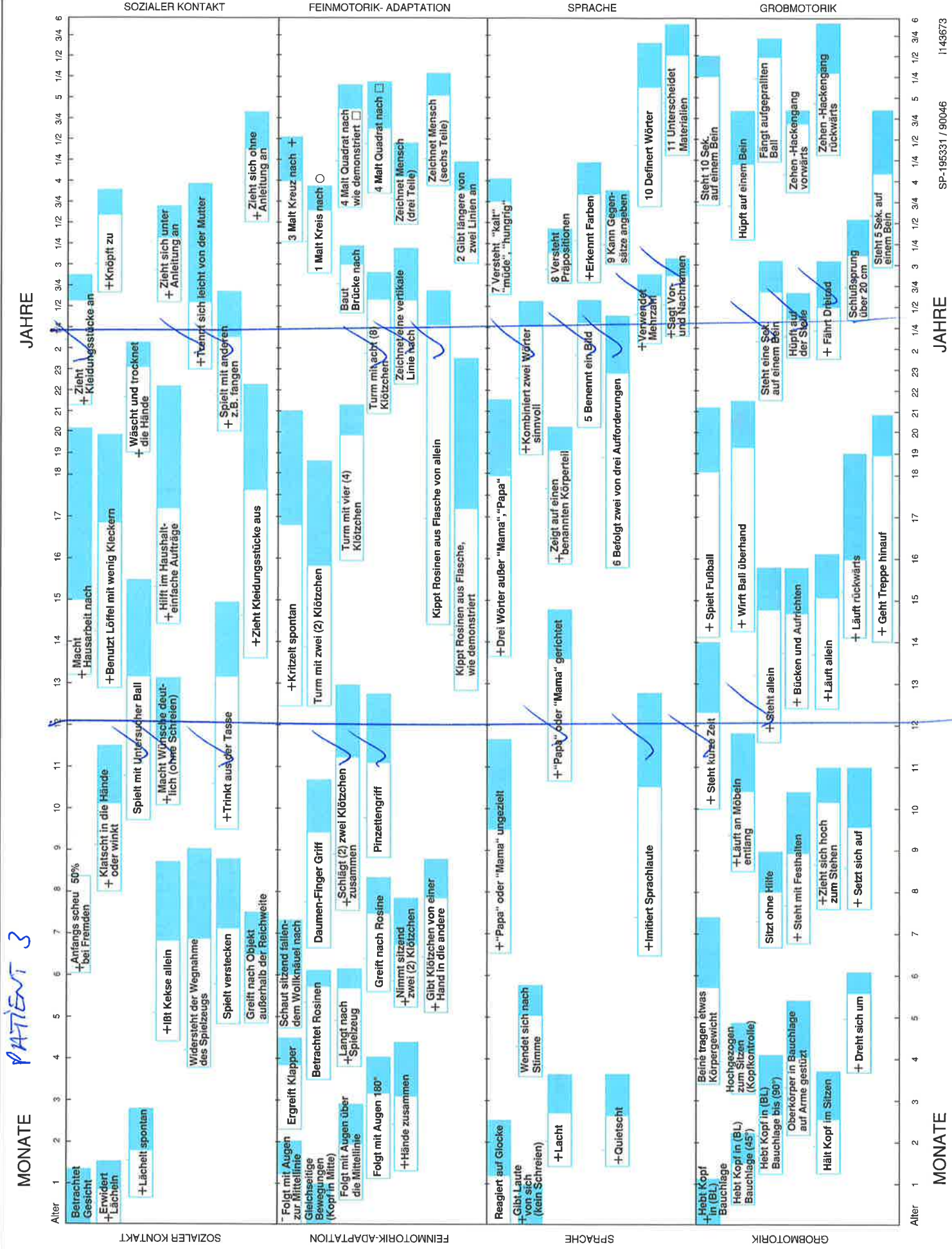

Supplement: Supplementary file 6 — Figure S6. Denver Developmental Screening Test results of patient 3. (PDF 198 kb) [file 12887_2019_1494_MOESM6_ESM.pdf]
