# Supplementary material for: First clinical experience with the Kora pacemaker system in congenital complete heart block in newborn infants
Source: BMC Pediatr. 2019 Apr 24;19:124. doi: 10.1186/s12887-019-1494-7 (PMC6480703; doi:10.1186/s12887-019-1494-7)
Supplement: Supplementary file 4 — Figure S4. Denver Developmental Screening Test results of patient 1. (PDF 198 kb) [file 12887_2019_1494_MOESM4_ESM.pdf]

Testanweisung beachten. Im Zweifelsfall bitte erneut informieren.

Name:

Geburtsdatum:

Datum der Untersuchung:

DENVER-ENTWICKLUNGSSKALEN

Bericht 25% 50% 75% 90%

Fußnote +2

(Siehe Rückseite)

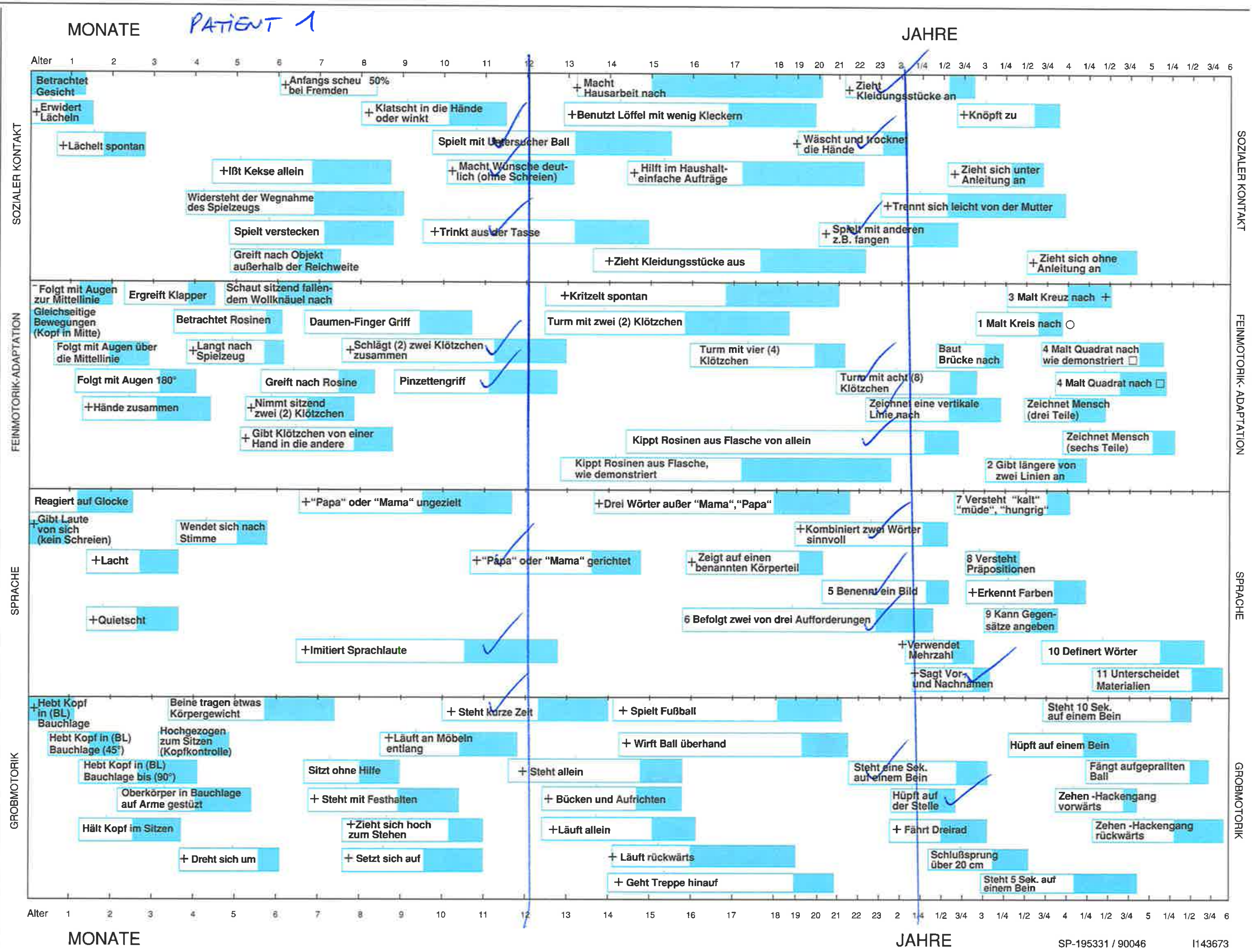

SP-195331 / 90046 I143673
