# Supplementary material for: First clinical experience with the Kora pacemaker system in congenital complete heart block in newborn infants
Source: BMC Pediatr. 2019 Apr 24;19:124. doi: 10.1186/s12887-019-1494-7 (PMC6480703; doi:10.1186/s12887-019-1494-7)
Supplement: Supplementary file 5 — Figure S5. Denver Developmental Screening Test results of patient 2. (PDF 202 kb) [file 12887_2019_1494_MOESM5_ESM.pdf]

Testanweisung beachten. Im Zweifelsfall bitte erneut informieren.

Name:

Geburtsdatum:

Datum der Untersuchung:

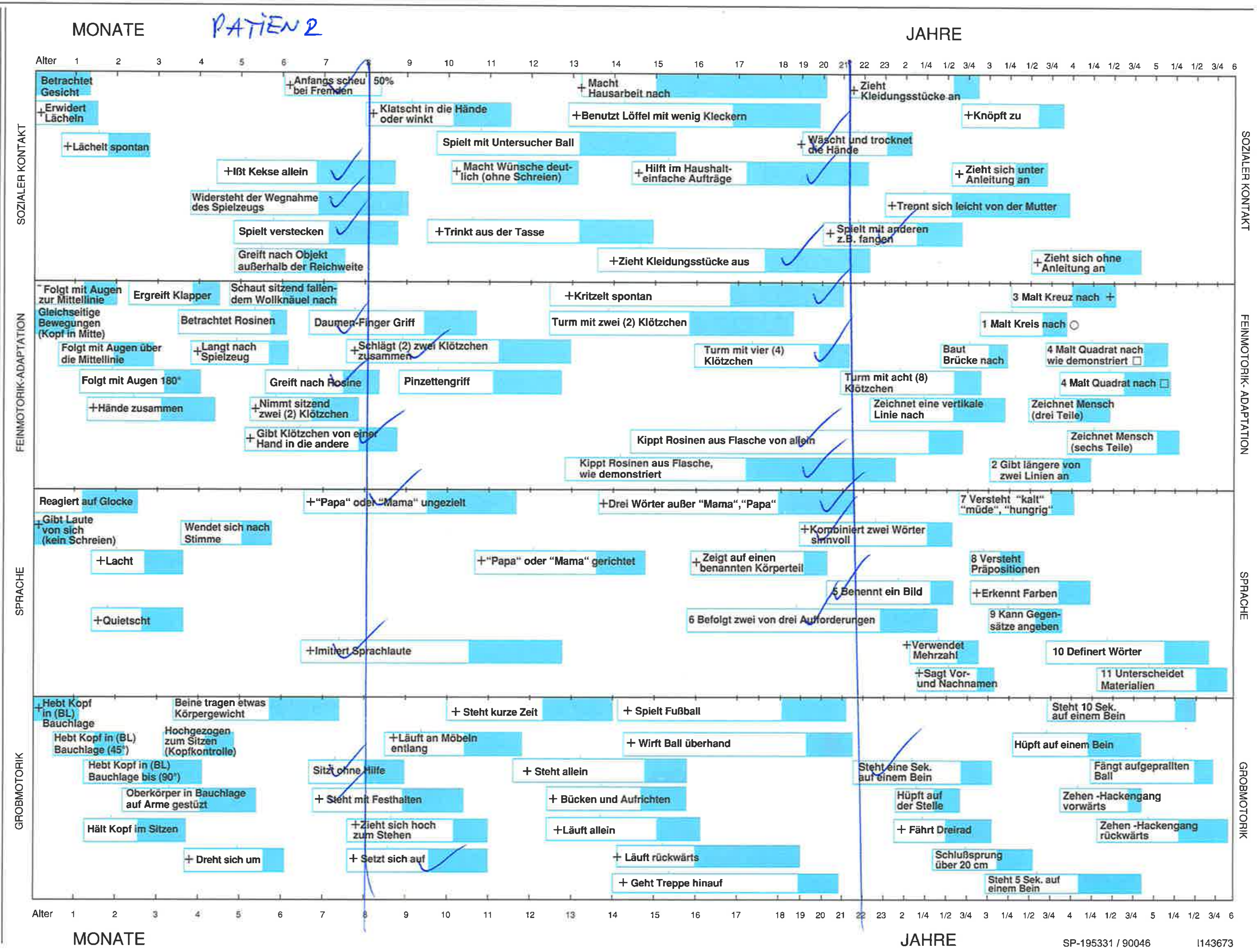

DENVER-ENTWICKLUNGSSKALEN

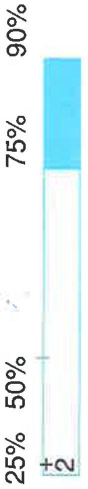

(Siehe Rückseite)

SP-195331 / 90046

I143673
